# Acceptability and Feasibility of Universal Offer of Rapid Point of Care Testing for HIV in an Acute Admissions Unit: Results of the RAPID Project

**DOI:** 10.1371/journal.pone.0035212

**Published:** 2012-04-27

**Authors:** Fiona Burns, Simon G. Edwards, Jeremy Woods, Golaleh Haidari, Yvette Calderon, Jason Leider, Stephen Morris, Rose Tobin, Jonathan Cartledge, Michael Brown

**Affiliations:** 1 Research Department of Infection & Population Health, University College London, London, United Kingdom; 2 Mortimer Market Centre, Central & North West London Foundation Trust, London, United Kingdom; 3 University College London Hospitals National Health Service Foundation Trust, London, United Kingdom; 4 Albert Einstein College of Medicine, Bronx, New York, United States of America; 5 University College London Centre of Applied Health Research, University College London, London, United Kingdom; 6 Department of Clinical Research, London School of Hygiene & Tropical Medicine, London, United Kingdom; Rush University, United States of America

## Abstract

**Background:**

UK guidance recommend all acute medical admissions be offered an HIV test. Our aim was to determine whether a dedicated staff member using a multimedia tool, a model found to be effective in the USA, is an acceptable, feasible, and cost-effective model when translated to a UK setting.

**Design:**

Between 14^th^ Jan to 12^th^ May 2010, a Health advisor (HA) approached 19–65 year olds at a central London acute medical admissions unit (AAU) and offered a rapid HIV point of care test (POCT) with the aid of an educational video. Patients with negative results had the option to watch a post-test video providing risk-reduction information. For reactive results the HA arranged a confirmatory test, and ensured linkage into HIV specialist care. Feasibility and acceptability were assessed through surveys and uptake rates. Costs per case of HIV identified were established.

**Results:**

Of the 606 eligible people admitted during the pilot period, 324 (53.5%) could not be approached or testing was deemed inappropriate. In total 23.0% of eligible admissions had an HIV POCT. *Of the patients who watched the video and had not recently tested for HIV, 93.6% (131/140) agreed to an HIV test*; four further patients had an HIV test but did not watch the video. Three tests (2.2%, 3/135) were reactive and all were confirmed HIV positive on laboratory testing. 97.5% felt HIV testing in this setting was appropriate, and 90.1% liked receiving the information via video. The cost per patient of the intervention was £21.

**Discussion:**

Universal POCT HIV testing in an acute medical setting, facilitated by an educational video and dedicated staff appears to be acceptable, feasible, effective, and low cost. These findings support the recommendation of HIV testing all admissions to AAU in high prevalence settings, although with the model used a significant proportion remained untested.

## Introduction

Late presentation to HIV services is the single most preventable cause of HIV related morbidity and mortality [1]. Late presentation also means a person must have been living with undiagnosed HIV infection for a substantial period of time, and people with undiagnosed HIV infection disproportionately contribute to onward transmission of the infection [2]. For these reasons, and for the associated increase in healthcare costs that late presentation brings [3], HIV prevention efforts have increasingly focused on improving opportunities to have an HIV test so as to reduce both late presentation and undiagnosed HIV infection [4]–[6].

The Jacobi Medical Center and North Bronx Healthcare Network (NBHN), New York, developed Project B.R.I.E.F. (**B**ehavior intervention, **R**apid HIV test, **I**nnovative video, **E**fficient cost and health care savings, **F**acilitated seamless linkage to outpatient HIV care). This program uses a “public health advocate”(PHA) to recruit stable patients attending an inner-city emergency department. It uses a model of universal testing with no specific population targeted. A multimedia tool (tablet PC) is used to deliver a validated video for HIV pre-test counselling (the video lasts ∼90 seconds and is available in 2 languages), followed by a rapid POCT. A post-test video, viewed while the individual waits for the test results, delivers risk reduction counselling and education. The educational videos are as effective as in-person counselling in conveying information related to testing [7]. Basic demographic and risk factor data are collected through this tool using touch screen technology. Using this model, of 7109 eligible patients who watched the video, 87% (6218) were tested, identifying 57 new infections [8].

The publication of the National guidelines on HIV testing [9] in 2008 led to a number of initiatives to assess the feasibility, acceptability and cost effectiveness of new models of delivery for HIV testing. Our aim was to determine whether a model of care utilising a multimedia tool and dedicated staff and found to be effective in an emergency medical setting in New York, is an acceptable, feasible and cost effective model in reducing late presentation of HIV infection when translated to a UK setting.

## Methods

### Ethics Statement

The local Research Ethics Committee, part of the National Research Ethics Service, waived requiring formal ethical approval under SL24 Project not considered to be research, version 4.0 April 2009, as they regarded the pilot as service evaluation.

Data was collected on all new admissions to an acute admission unit (AAU) in Central London over a 16 week period (15^th^ Jan to 11^th^ May 2010 inclusive). Adults aged 19–65 in a stable clinical condition were eligible for inclusion in the HIV testing pilot. Patients who were only on the AAU during the weekend when the HIV testing service was not available were excluded from this analysis.

The service model employed consisted of a health advisor (HA) approaching all new stable admissions, and offering HIV testing with the aid of an educational video available in up to 4 languages. If patient accepted, a finger prick rapid HIV point of care test (POCT) was performed using the EU approved INSTI™ test (sensitivity and specificity reported elsewhere) [10]. If the result was HIV negative the patient had the option of watching a post-test video providing risk-reduction information. If the result was reactive the HA explained the need for a confirmatory test, arranged the test and urgent follow up with the HIV service. All patients watching either video completed a questionnaire designed to evaluate patient satisfaction and collect process evaluation data (demographic and behavioural profiles). The questionnaire was delivered electronically with touch-screen technology via the tablet PC that patients used to watch the videos.

The video scripts were adapted from those used in Project BRIEF to suit a UK context and to meet BHIVA guidelines [9]. They were pretested in two focus groups with service providers and users. At the time of the pilot only an audio English version was available but now versions exist with English, French, Polish, and Spanish subtitles.

The tablet PC and all equipment necessary for the POCT tests were located on a small portable trolley that was wheeled to the bedside. Disposable headsets were used so as not to disturb other patients.

Following the completion of the project all staff who worked on the unit during the pilot period were surveyed about their experiences. Surveys were distributed at meetings for those still working on the unit and via email to those now located elsewhere.

Standard statistical tests, e.g. Chi^2^ test and Student’s t-test, were used to examine associations between variables.

We directly calculated the incremental cost of the educational video intervention versus treatment as usual from a National Health Service (NHS) perspective. The cost components included in the analysis were video equipment costs, cost of disposable equipment and staff costs incurred through training, delivering the intervention and post-test counselling. Resource use data for each cost component were collected in the study. We applied unit costs from market prices and published sources [11]. We conservatively assumed that the lifetime of the video equipment was approximately 1000 patients. The majority of the costs incurred were staff costs. We assumed in our main analysis that these would be incurred by a Health Advisor (Band 7), based on 3 tests per hour. In sensitivity analyses we explored the impact of using different staff and increasing the number of tests per hour.

## Results

During the study period there were 606 eligible (19 to 65 years of age inclusive and inpatient on weekday) admissions to the AAU, representing 602 individuals. As none of the repeat attendees had an HIV test on their first visit both visits are included in subsequent analyses. Three quarters (456/606, 75.3%) of all eligible admissions were approached to participate in the study. There were no significant differences in the gender, age, ethnicity, presentation pattern or length of stay between those approached and not approached (table 1).

**Table 1:** Characteristics of eligible patients admitted to AAU during pilot.

Characteristic	Total (N = 606)%	Approached (n = 456)%	P value
Male	56.8	56.6	0.892
Age (years) (median age 44)			0.126
19–35	32.7	34.4	
36–65	67.3	65.6	
Ethnicity (n = 590)			0.726
British	42.4	42.7	
African	4.7	4.3	
Other/not stated	52.9	53.0	
Indicator disease* present during admission (n = 591)	13.5	13.2	0.695
Inpatient <48 hours	53.9	53.6	0.842

* As defined in National Guidelines on HIV testing [9].

Despite often multiple attempts, over half (53.5%) of approaches failed to encounter or engage the patient (table 2). Of the 282 patients whom were asked if they would be involved in the pilot project, 153 (54.3%) agreed. On introduction of video four patients asked or agreed to have an HIV test but did not want to watch the video, and five disclosed that they had recently tested for HIV and therefore withdrew from further involvement. Following the video a further eleven patients declined to test: four had tested within the past three months; two had never been sexually active; two declined because of communication difficulties; one wanted to test in an anonymous environment and was referred to the local sexual health clinic; one became unwell during the video; and one declined. In all, of the 140 patients who watched the video and had not tested for HIV in the preceding three months, 93.6% (131/140) agreed to an HIV test. All patients tested received their results at the time of testing.

**Table 2:** Outcome to bedside approach.

Of 606 eligible admissions:	N (%)
Known HIV positive	7 (1.2%)
Patient discharged	44 (7.3%)
Patient absent	64 (10.6%)
Patient too unwell^1^	107 (17.7%)
Unable to consent^2^	56 (9.2%)
Other^3^	38 (3.0%)
Tested already^4^	8 (1.3%)
Consent sought	282 (46.5%)

1 Ward staff provided information on who should not be approached due to ill health on daily basis.

2 Usually due to intoxication or psychiatric illness.

3 Includes relatives or friends visiting, eating, language barriers and with staff.

4 HIV test already performed during current admission.

Older people (aged 40 years or more) were less likely to accept to watch the video than younger patients (43.4% vs. 64.2%, p  =  0.001), however if they did so uptake of the test did not differ by age (71.8% vs. 76.9%, p  =  0.476). There was no difference in the uptake of the video or HIV test by gender.

In total 23.0% of eligible admissions to AAU during the pilot period had a POCT HIV test, and 25.7% left AAU knowing their HIV status having tested on that admission or within the preceding three months, or previously been diagnosed HIV positive. Three tests (2.2%, 3/135) were reactive on POCT and all were confirmed HIV positive on further laboratory testing.

The three patients diagnosed HIV positive were a 48 year old British man with pneumonia, presumed *Pneumocystis jirovecii* pneumonia (PCP), CD4 20; a 42 year old Nigerian woman admitted with bacterial pneumonia, CD4 40; and a 60 year old British man with rectal bleeding on warfarin, CD4 590. All three patients were seen by specialist HIV services whilst an inpatient and remain engaged with HIV services 12 months on. Only one of the three had previously tested for HIV, the 48 year old British man having tested over 5 years previously.

### Respondent Characteristics

The majority of RAPID participants (patients who watched the video and completed the survey) were male (58.6%), and the median age was 38.5 years. Over half (51.9%) resided in the hospital catchment area (local boroughs of Camden or Islington), and 85.5% were from within London. Over two fifths (42.8%) of participants were born abroad: 19 (12.5%) in Europe, 17 (11.2%) in Africa (9, 5.9% black African) and 15 (9.9%) in Asia or the Indian sub-continent. During the pilot the video was only available in spoken English (subsequently versions with English, French, Polish and Spanish subtitles have been developed), despite this 87.5% of patients stated the video was in their preferred language.

Forty percent (61/152) of participants had previously had an HIV test, however only 22 (14.5%) had tested within the past 12 months and 19 (12.5%) had tested more than 5 years previously. Five participants who had previously tested (14.8%, 5/61) reported they never received the results of their last HIV test. Almost 20% of participants reported behaviour associated with increased risk for HIV (table 3). Prior HIV testing was more prevalent in those reporting an HIV risk behaviour than those who did not (75.0% vs. 32.8%, p<0.001).

**Table 3:** Reporting of HIV risk factors by gender (n = 147).

Ever reported:	Total (N = 147)	Men (N = 85)	Women (N = 62)
	% (n)	% (n)	% (n)
Previous STI	9.5% (14)	11.8% (10)	6.5% (4)
Injecting drug use	1.4% (2)	2.4% (2)	0
Sex with a man who has sex with men	7.5% (11)	10.6% (9)	3.2% (2)
Sex with an HIV positive person	1.4% (2)	1.2% (1)	1.6% (1)
Sex with a person who uses injection drugs	2.7% (4)	3.5% (3)	1.6% (1)
None of the above	81% (119)	76.5% (65)	87.1% (54)

### Patient Acceptability

The overwhelming majority (97.5%) of participants of the RAPID pilot thought POCT HIV testing in the AAU was a good idea, and 96.7% thought rapid HIV testing appropriate in this setting. Almost all (90.1%, 137/152) participants liked receiving information via video, and the majority (80.3%, 133/152) felt the short video answered their questions about HIV testing. The main reason for patients not liking the use of video was because they would prefer face to face interaction (11/14); two patients also mentioned that they were not able to ask questions they wanted to, and another two felt the leaflet introducing the project had already provided sufficient information. Participants were asked if the Health advisor running the pilot made it easy to get tested: 82.9% responded ‘yes, very’, 10.5% ‘yes, a little’, and 5.9% ‘not sure’.

Finally participants were asked how they would prefer rapid HIV testing to be offered: 2.6% preferred video alone, 15.8% a health advisor alone, 9.2% a combination of health advisor and leaflet, 46.1% the combination of video and health advisor, and 25.7% a combination of health advisor, video and leaflet.

### Acceptability to Staff

Responses were obtained from 61.5% (88/143) of clinical staff working on AAU during the pilot; the response rate was lower amongst doctors of all grades (50%), than among nurses and health care assistants (74.1%). No staff felt the service had disrupted their job in any way, and all staff felt the service should be continued. 92% of doctors believed that more of their own patients were now tested for HIV (table 4), and no doctors felt the service made them less likely to offer a test; with three-quarters of doctors believing the service increased the likelihood of them requesting an HIV test either directly or via the service.

**Table 4:** Staff attitudes and experiences of RAPID project.

	Doctors (n = 44)	Nurses & Health-care Assistants (n = 40)
Aware of service	93%	85%
Is this service:		
Not needed	–	–
Useful	30%	21%
Very useful	60%	38%
No opinion	10%	11%
Influence of a dedicated person offering HIV tests on the number of own patients having anHIV test:		
More people now tested	92%	85%
Less people now tested	3%	–
No change in number tested	5%	15%
Influence of having a dedicated HIV testing service on requesting an HIV test from patients		
More likely to offer test directly	35%	Not applicable
More likely to ask RAPID service to offer test	40%	Not applicable
Less likely to request test as assume will occur as part of RAPID	–	Not applicable
Not changed my practice	25%	Not applicable

Regular POCT testing on AAU continued until June 25^th^, and the number of standard laboratory HIV test requests in AAU in the five months preceding and during pilot did not significantly differ (data not shown).

### Cost

The additional cost of the equipment required for the educational video was £1709 (Table 5). The incremental cost of the education video intervention per patient was £21 (Table 6). The largest component of the cost was the staff cost to run the video and test and associated administration (49% of the total incremental cost). The cost per case identified was £1,083. If the costs of disposable equipment were excluded on the basis that these would have been incurred in any case, then the incremental cost of the education video per patient fell from £21 to £15. If the service was provided by a Nurse Band 5 rather than a Health advisor Band 7 the cost per patient fell from £21 to £18. If it was provided by a Healthcare Assistant they fell to £14. If 6 rather than 3 tests were undertaken per hour then the costs per patient were £16, £14 and £12, depending on whether the staff member involved was a Health advisor Band 7, Nurse Band 5 or Healthcare Assistant.

**Table 5:** Equipment costs.

Equipment	Cost (ex VAT)^1^
	£
**Start up costs** (excluding video production^2^)	
Toshiba Portege M750-13c T5870^3^	979
Simple-smart Laptop Cart^4^	325
Kensington Micro Saver Disc Lock^3^	29.99
Sharps bin	1.73
SNAP Survey software license	373.75
Total:	1709.47
**Disposables** (per test performed)	
INSTi kits	6.50
Disposable Gloves	0.05
Lightweight Stereo Headphones	0.88
Pulp tray	0.03
Total:	7.46

1 As bought in December 2009.

2 The videos are available free of charge from corresponding author so this cost would not need to be replicated if same service was to be implemented elsewhere.

3 Supplied by Misco.

4 Supplied by RDP Health.

**Table 6:** Cost estimates for first 1000 patients.

Activity		Health advisor Band 7	Nurse Band 5	Health-Care Assistant
Cost per hour^1^		£36	£26	£14
Training		£1,350.00	£975.00	£1,050.00
		(37.5 hours)	(37.5 hours)	(75 hours)
Start up costs^2^ (see table 5)		£1,709.47	£1,709.47	£1,709.47
**3 tests per hour**				
Watched video but no test	11.7 hours	£421.20	£304.20	£163.80
Withdraws as recently tested - no video	1.13 hours	£40.68	£29.38	£15.82
Tests for HIV but does not watch video	4.33 hours	£155.88	£112.58	£60.62
Watches video & tests for HIV	290 hrs	£10,440.00	£7,540.00	£4,060.00
Post test counselling (reactive tests only) &linkage to care	14.8 hours	£532.80	£384.80	£207.20
Disposables	896 tests	£6,684.16	£6,684.16	£6,684.16
Total:		£21,334.19	£17,739.59	£13,951.07
Cost per patient		£21.33	£17.74	£13.95
Cost per case identified		£1,082.95	£900.49	£708.18
**6 tests per hour** 2				
Watched video but no test	5.85 hours	£210.60	£152.10	£81.90
Withdraws as recently tested -no video	1.13 hours	£40.68	£29.38	£15.82
Tests for HIV but does not watch video	4.33 hours	£155.88	£112.58	£60.62
Watches video & tests for HIV	145 hours	£5,220.00	£3,770.00	£2,030.00
Post test counselling (reactive tests only) &linkage to care	14.8 hours	£532.80	£384.80	£207.20
Disposables	896 tests	£6,684.16	£6,684.16	£6,684.16
Total:		£15,903.59	£13,817.49	£11,839.17
Cost per patient		£15.90	£13.82	£11.84
Cost per case identified		£807.29	£701.40	£600.97

1 Costs including qualifications. Taken from ‘Unit Costs of Health & Social Care 2010’ [11].

2 Refer to table 5. Most equipment would be expected to last well beyond the first 1000 patients so future costs would reduce accordingly.

3 Would require either simultaneous use of two laptops (parallel testing) or not including patient survey (collection of data on patient demographics, risk profile, acceptability). If parallel testing need to add further £1335.72 to start up costs (for second work station).

## Discussion

Routine HIV point of care testing in the AAU setting was acceptable to patients. It was successful in identifying cases of HIV and demonstrates the potential for earlier diagnosis in screening those without indicator diseases. Using a service model with a dedicated staff member compared to one where HIV testing is embedded within routine clinical practice undoubtedly increased costs, however the cost per case identified still compares favourably with other screening programmes in the UK [12]. The provision of a dedicated staff member also ensured staff acceptability and no disruption to current services.

For this pilot we deliberately used a senior Health Advisor with a lot of experience in delivering HIV results however with appropriate training and support a more junior staff member could run the service. In project BRIEF in New York, this role is fulfilled by ‘Public Health Advocates’, who usually have no formal clinical training other than the two weeks of training they get once employed on the project.

The use of digital media (the video) ensured consistent messaging, and that information was conveyed in an easy manner for patients with health literacy or literacy issues. Digital media has the ability to overcome linguistic issues, to be flexible around patient care, and can be delivered on sustainable system wide tools, for example patient television. Furthermore the use of video was liked by patients although the survey data suggests that face to face contact time remains important. Unfortunately different models of service delivery, such as video or health advisor alone, were not able to be assessed in the current study.

For the purposes of this paper costings are based on the first 1000 patients seen however the video equipment would be expected to last well beyond the first 1000 patients and subsequent costs would reduce accordingly. 22% of all patients aged 19–65 years were admitted to the AAU and subsequently discharged between 5 pm Friday and 9 am Monday and thus excluded from this study. These patients were likely to be younger (mean age 40 versus 44 years) but there was no gender difference compared with patients on AAU during testing hours. The authors would expect more alcohol related admissions over weekends but whether this directly equates with HIV risk is unknown.

The main criticism of this model could be that it fails to embed HIV testing within routine clinical practice, often referred to as ‘normalising HIV’ [13]. This is a concern that the authors share, however without the will of clinical colleagues in other specialties, sustained routine HIV testing within general medical practice in the UK currently appears to elude us; the notable exception to this of course being the hugely successful universal antenatal screening programme [14]. Implementation of the HIV antenatal screening programme was supported by specific national health policy [15]. While guidelines have been published recommending expansion of HIV testing opportunities to setting such as AAUs these fall short of policy recommendations.

A further criticism could be that two of the three cases identified were likely to have been detected through the already locally established practice of targeted testing of high risk individuals as defined by indicator disease or risk group [16], and that expanding practice to universal testing is unnecessary. The authors would certainly like to believe that the two cases with indicator diseases would have been identified without the RAPID pilot in place, unfortunately published data suggests that this may not necessarily have occurred [16], [17]. Earlier diagnosis is increasingly acknowledged as fundamental in prevention of HIV related morbidity and mortality, and in the prevention of onward transmission of infection. Testing based on indicator diseases, as found in the current study, is unlikely to identify people in the early stages of HIV infection. The third case, the 60 year-old gentleman admitted with rectal bleeding, reported no high risk behavior and to our knowledge had never accessed sexual health services; without universal testing his HIV would almost certainly have remained undetected until presenting at some later date with advanced disease.

No difference was found between those approached and not approached in terms of gender, ethnicity, patient stay, or indicator disease status, suggesting the pilot used a truly non targeted approach. Generalisability to other clinical settings however is hindered by the highly specific population and setting of the study. HIV case-finding outside of the London AAU setting is likely to influence the cost-effectiveness of the model.

For any screening procedure to be viable it must fulfill certain criteria, often called the Wilson-Jungner criteria [18]. The criteria are listed in short below followed by findings based on current study.*Wilson-Jungner Screening Criteria and HIV testing in AAUs.*

- *The condition that is being sought must be sufficiently common in the group being screened to make screening worthwhile.*

Taking those already known to be HIV positive (n = 7) and those newly diagnosed (n = 3) the diagnosed HIV positive prevalence in the AAU setting was 1.7% (10/606) – suggesting AAU is a highly appropriate location to target HIV testing in areas of high prevalence.

- *Screening will lead to earlier detection of a treatable disease so that outcome is significantly improved.*

Universal screening has lead to early detection in one of the three cases identified. Earlier detection of HIV is associated with reduced short term and all cause mortality [19], [20].

- *The screening technique has acceptable costs per case identified and the procedure is acceptable to both patients and staff.*

Even at the highest cost estimate of £1083 per case identified, this program demonstrated reasonable cost and acceptability to staff and patients.

- *There will be a high enough uptake to make the procedure valid.*

Uptake of the POCT test was extremely high once patients watched the video. Alternative strategies such as universal offer of POCT HIV test in the absence of video were not explored in the current pilot.

- *There will be high specificity (low rate of false positives) and a very high sensitivity (very low rate of false negatives).*

The specificity of the test is marketed at 99.5% and the sensitivity at 97.5% [10].

- *Investigation and management of positive results will not overburden the system.*

Having a dedicated staff member minimizes potential disruption to services. They can arrange confirmatory testing, post test counselling, and linkage into HIV care. All test results are delivered at the time of testing.

### Implications

It is estimated that prevention of one new HIV infection in the UK saves between £280,000 and £360,000 in direct lifetime healthcare costs [21]. Thirty five Primary care Trusts in England have a prevalence of diagnosed HIV greater than 2 per 1,000 adult population. Whilst failing to embed HIV testing within routine clinical practice, utilization of a model of universal POCT HIV testing in an acute medical setting, facilitated by an educational video and dedicated staff may play an important role in the transition to normalization of HIV testing, as this model appears to be acceptable to both staff and patients, feasible, effective, and cost-effective.

With minimal staff training this model could also be adapted to one of universal POCT testing within routine clinical care. A clearly identified pathway to link those with reactive tests into specialist care for confirmatory testing, post test counselling, and linkage into care should ideally support any such initiative, ideally through the provision of a HIV liaison nurse/health advisor.
